# Chikungunya Virus Overcomes Polyamine Depletion by Mutation of nsP1 and the Opal Stop Codon To Confer Enhanced Replication and Fitness

**DOI:** 10.1128/JVI.00344-17

**Published:** 2017-07-12

**Authors:** Bryan C. Mounce, Teresa Cesaro, Lea Vlajnić, Anna Vidiņa, Thomas Vallet, James Weger-Lucarelli, Gabriella Passoni, Kenneth A. Stapleford, Jean-Pierre Levraud, Marco Vignuzzi

**Affiliations:** a Institut Pasteur, Viral Populations and Pathogenesis Unit, CNRS UMR 3569, Paris, France; b Institut Pasteur, Macrophages and Development of Immunity Unit, CNRS UM3738, Paris, France; Washington University School of Medicine

**Keywords:** antiviral, antiviral resistance, chikungunya virus, polyamines, viral replication

## Abstract

Polyamines, which are small positively charge molecules present in all cells, play important roles in the replication of DNA and RNA viruses. Chikungunya virus (CHIKV) relies on polyamines for translation of the viral genome upon viral entry, and pharmacological depletion of polyamines limits viral replication. However, the potential development of antiviral resistance necessitates a better understanding of how polyamines function and can be targeted via compounds that alter polyamine levels. We have isolated CHIKV that is resistant to polyamine depletion and contains two mutations in the nonstructural protein 1 (nsP1)-coding region in combination with a mutation to the opal stop codon preceding nsP4. These mutations, in addition to promoting viral replication in polyamine-depleted cells, confer enhanced viral replication *in vitro* and *in vivo*. The nsP1 mutations enhance membrane binding and methyltransferase activities, while the stop codon mutation allows increased downstream translation. These mutations, when combined, enhance viral fitness, but individual mutants are attenuated in mosquitoes. Together, our results suggest that CHIKV can evolve resistance to polyamine depletion and that pharmaceuticals targeting the polyamine biosynthetic pathway may be best used in combination with other established antivirals to mitigate the development of resistance.

**IMPORTANCE** Chikungunya virus is a mosquito-borne virus that has infected millions worldwide. Its expansion into the Americas and rapid adaptation to new mosquito hosts present a serious threat to human health, which we can combat with the development of antiviral therapies as well as understanding how these viruses will mutate when exposed to antiviral therapies. Targeting polyamines, small positively charged molecules in the cell, may be a potential strategy against RNA viruses, including chikungunya virus. Here, we have described a virus that is resistant to polyamine depletion and has increased fitness in cells and in full organisms. Mutations in viral genome capping machinery, membrane binding activity, and a stop codon arise, and their altered activities enhance replication in the absence of polyamines. These results highlight strategies by which chikungunya virus can overcome polyamine depletion and emphasize continued research on developing improved antiviral therapies.

## INTRODUCTION

Chikungunya virus (CHIKV) is an arthropod-borne virus (arbovirus) from the Alphavirus family that has caused several severe outbreaks worldwide. Clinical manifestations can be severe and long lasting, including fever and chronic joint pain, and rapid spread of the virus into new territories underscores its threat to human health. Currently, no antiviral treatment is available, though vaccine candidates hold promise (1–3). Its sweeping entry into the Americas in 2013 underscored its ability to rapidly infect new locations (4), and since that time, the virus has established itself within the region. Additionally, mutations within the viral genome, particularly in its envelope protein E1, allowed the virus to gain fitness in Aedes albopictus mosquitoes, expanding the repertoire of hosts for the virus (5, 6). Given the alarming recent spread of RNA viruses, including arboviruses such as CHIKV and Zika virus, the development of novel therapeutics to combat viral replication is necessary.

Polyamines are carbon chains with positively charged amino groups, found within all cell types, that are associated with multiple cellular functions, including transcription, translation, DNA replication, and signaling, among others (7). Via the drug difluoromethylornithine (DFMO), polyamine biosynthesis can be blocked and polyamines subsequently depleted through the inhibition of ornithine decarboxylase, the key enzyme in polyamine biosynthesis (8). In addition to their roles within the cell, polyamines are crucial for replication of RNA viruses, including CHIKV. We previously reported that CHIKV relies on polyamines at the levels of translation and RNA-dependent RNA polymerase activity in infected cells (9). In fact, diverse RNA viruses rely on polyamines for their replication both *in vitro* and *in vivo* (10, 11). Thus, targeting polyamines holds promise for the development of broad-spectrum antivirals, including for CHIKV.

RNA viruses have error-prone polymerases and thus mutate rapidly, including in the presence of antiviral molecules (12). The development of antiviral resistance necessitates the development of novel therapeutics to combat viral infection. However, being able to anticipate the emergence of resistant viruses also provides valuable information concerning the feasibility of using antivirals clinically. Understanding the molecular determinants of resistance can provide new insights into viral replication and pathogenesis, as well as drug action. By further exploring the mechanisms of viral replication and the drugs that target these processes, better therapeutics can be rationally designed to enhance antiviral activity and prevent resistance from developing.

By passaging virus on DFMO-treated cells, we generated CHIKV that was resistant to polyamine depletion. Following the isolation of these CHIKV mutants resistant to polyamine depletion *in vitro*, we identified three unique mutations that confer resistance when present in combination: two in nonstructural protein 1 (nsP1) and one that changes the opal stop codon between nsP3 and nsP4 into arginine. CHIKV with either mutation individually was not resistant to polyamine depletion and exhibited growth defects in mosquitoes. Viruses with combinations of mutations had enhanced replication and fitness *in vitro* and *in vivo* in both mammalian and mosquito systems. We found that one nsP1 mutation enhances membrane binding, while another mutation promotes genome methylation. The stop codon mutation allows for increased downstream translation. Together, these mutations promote viral replication. Our data show that CHIKV rapidly adapts to polyamine depletion via novel mutations that confer enhanced viral replication and fitness.

## RESULTS

### Passage of CHIKV in DFMO confers resistance to polyamine depletion.

**FIG 1:**
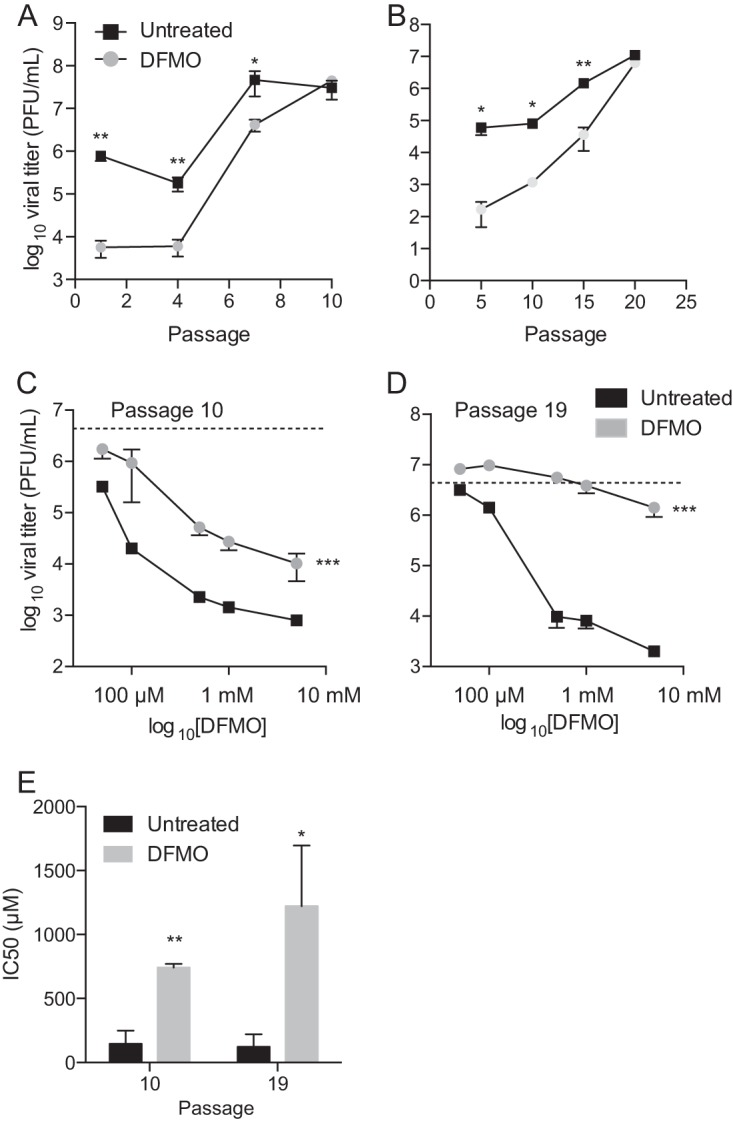
Passage of CHIKV in DFMO-treated cells confers resistance to polyamine depletion. (A) CHIKV titers across passages at a fixed MOI (0.1) in BHK-21 cells treated with 500 μM DFMO for 4 days prior to infection. (B) CHIKV titers across blind passages in BHK-21 cells treated with DFMO. (C and D) BHK-21 cells were treated with increasing doses of DFMO and infected with virus passaged 10 times (C) or 19 times (D) with or without DFMO. Dashed lines represent the average titer of virus from infection of untreated cells. (E) IC_50_ values of viruses from blind passages 10 and 19. *, *P* < 0.05; **, *P* < 0.01 (using Student's *t* test; *n* = 3). Error bars represent one standard error of the mean.

To determine how CHIKV may adapt to a polyamine-depleted environment, we passaged virus in BHK-21 cells that were treated with 500 μM DFMO (or left untreated for controls) for 4 days prior to infection, which we demonstrated previously to inhibit CHIKV replication but not alter cell viability or cycling (9). After each passage, viral titers were determined and used to infect the next passage at a constant multiplicity of infection (MOI) of 0.1. Over the course of passaging the virus, viral titers increased for virus passaged both in untreated cells and in DFMO-treated cells, and the titers of viruses passed in DFMO-treated cells reached untreated levels in 10 passages (Fig. 1A). In addition, passages were done without checking viral titers prior to the next passage (blind passaging), and, as with fixed-MOI passages, viral titers increased through the passages, and virus from DFMO-treated cells reached untreated levels in 20 passages (Fig. 1B). To test whether these viruses were resistant to DFMO, we treated cells with increasing doses of DFMO, from 50 μM to 5 mM. At both passages 10 and 19 of the blind passages (Fig. 1C and D), viruses passaged in the presence of DFMO exhibited reduced sensitivity to the drug compared to wild-type virus (squares) and compared to untreated controls (dashed lines), especially at passage 19, when the viruses exhibited resistance even at the highest dose. Fifty percent inhibitory concentration (IC_50_) values were calculated from these curves via GraphPad Prism's built-in statistical analysis, and while the virus passaged in untreated cells maintained the same level of sensitivity, virus passaged in DFMO-treated cells exhibited a 10- to 20-fold increase in IC_50_ (Fig. 1E).

### Mutations of nsP1 and the opal stop codon confer resistance to polyamine depletion in mammalian cells.

Following passage, viruses were sequenced to determine mutations present within the populations. Viruses from passage 10 of fixed-MOI passages and passage 19 of blind passages were sequenced, including untreated controls. Sequences were aligned to the wild-type CHIKV sequence, and mutations were identified when detected in the alignment and confirmed by comparison with peaks on chromatograms. We observed three distinct mutations emerge in the blind passages, namely, G230R and V326M mutations in nsP1 and a change of the opal stop codon into an arginine (here named XR). These mutations were present in all three independent replicates of the viral passages but not in the untreated controls (Table 1). We observed several other mutations at the consensus level, but the mutations were not consistent among the replicates of the passages. We found no consensus-level mutations in the untreated samples, though minority variants were not investigated. Similar mutations were found in the fixed-MOI passages, though the G230R mutation was not detected (Table 2).

**TABLE 1:** Mutations detected after 19 blind passages of CHIKV in 500 μM DFMO^*a*^

Triplet change	Amino acid change	Protein	Virus(es)^*b*^
**GGG→AGG**	**G230R**	**nsp1**	**DFMO 1, 2, 3**
**GTG→ATG**	**V326M**	**nsp1**	**DFMO 1, 2, 3**
GAG→AAG	D252N	nsp2	DFMO 2
GAG→AAG	E253K	nsp2	DFMO 2; NT 1, 3
GGG→AGG	G117R	nsp3	DFMO 3
GAC→AAC	D119N	nsp3	DFMO 1
**TGA→CGA**	**X→R**	**nsp3**	**DFMO 1, 2, 3**
GAG→GAT	E227D	nsp4	DFMO 1
TGT→GGT	C228G	nsp4	DFMO 1
ACT→CCT	T298P	nsp4	DFMO 1
GTG→GGA	V297G	nsp4	DFMO 1
ACA→ATA	T58R	E2	DFMO 3
GCA→CCA	A89P	E2	DFMO 2
CCG→CGG	P90R	E2	DFMO 2

a Boldface indicates mutations examined in this study.

b Independent passages differentiated by number and treatment. NT, not treated with DFMO.

**TABLE 2:** Mutations detected after 10 fixed-MOI passages of CHIKV in 500 μM DFMO^*a*^

Triplet change	Amino acid change	Protein	Virus(es)^*b*^
AAA→GAA	K234E	nsp1	NT 1
**GTG→ATG**	**V326M**	**nsp1**	**DFMO 1, 2, 3**
**TGA→CGA**	**X→R**	**nsp3**	**DFMO 1, 2, 3**
ATC→ACC	I54T	E2	DFMO 1
AAG→AAC	K67N	E2	DFMO 2
GCA→CCA	A89P	E2	NT 1
CCG→CGG	P90R	E2	NT 1
GGT→GGA	Silent (G)	E1	DFMO 3

a Boldface indicates mutations examined in this study.

b Independent passages differentiated by number and treatment. NT, not treated with DFMO.

**FIG 2:**
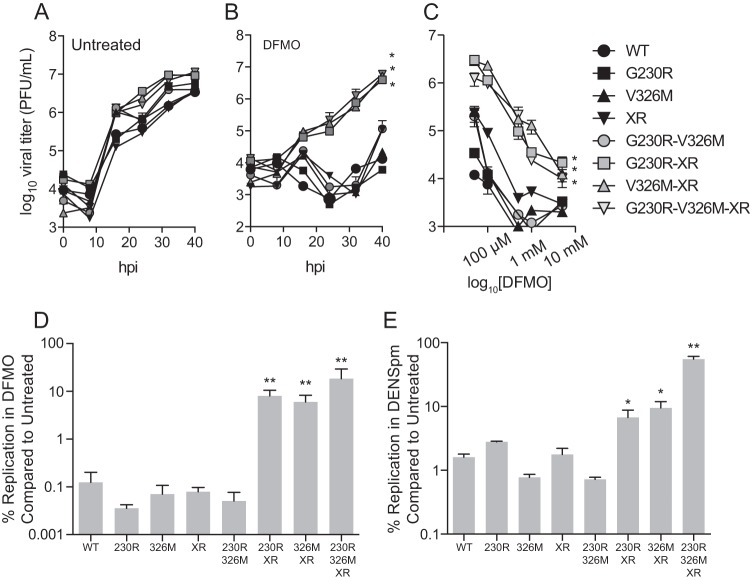
Combination of nsP1 and stop codon mutations confers resistance to polyamine depletion. (A and B) BHK-21 cells were left untreated (A) or treated with 500 μM DFMO for 4 days (B) and subsequently infected with CHIKV, and viral titers were determined by plaque assay at the indicated times postinfection. (C) BHK-21 cells were treated with increasing doses of DFMO prior to infection with CHIKV. Titers were determined at 24 hpi. (D and E) BHK-21 cells treated with 500 μM DFMO (D) or Vero-E6 cells treated with 50 μM DENSpm (E) were infected with CHIKV, and titers were determined at 24 hpi. The percentage surviving treatment was calculated by dividing titers from treated cells by titers from untreated cells. *, *P* < 0.05; **, *P* < 0.01 (using Student's *t* test; *n* = 3). Error bars represent one standard error of the mean.

To test the effect of the mutations identified in our passages, we added the G230R, V326M, and XR mutations to CHIKV by site-directed mutagenesis, singularly and in combination. These viruses replicated well in BHK-21 cells (Fig. 2A); the G230R-XR, V326M-XR, and G230R-V326M-XR mutants (gray symbols) exhibited a 4- to 10-fold increase in titers. Under DFMO treatment conditions, none of the single mutants replicated, and the G230R-XR, V326M-XR, and G230R-V326M-XR mutants replicated with slightly delayed kinetics (Fig. 2B, gray symbols). Cells treated with increasing doses of DFMO were infected with all viruses, and the same double and triple mutants exhibited resistance to polyamine depletion (Fig. 2C). In fact, at a dose of 500 μM DFMO, the G230R-XR, V326M-XR, and G230R-V326M-XR mutants exhibited 10% survival compared to untreated controls, whereas the other viruses had survival of around 0.1% (Fig. 2D). DFMO functions by blocking the synthesis of polyamines by inhibiting ornithine decarboxylase (13). Another molecule that depletes cells of polyamines is diethylnorspermine (DENSpm), which acts by upregulating the expression of spermine-spermidine acetyltransferase 1 (SAT1) to acetylate and deplete spermidine and spermine (14). As we previously reported, CHIKV was sensitive to DENSpm (9), and the same three mutants that we found to be resistant to DFMO were also resistant to DENSpm (Fig. 2E), implicating these mutations as being crucial for replication in polyamine-depleted cells.

### Resistant viruses exhibit fitness enhancement *in vitro*.

**FIG 3:**
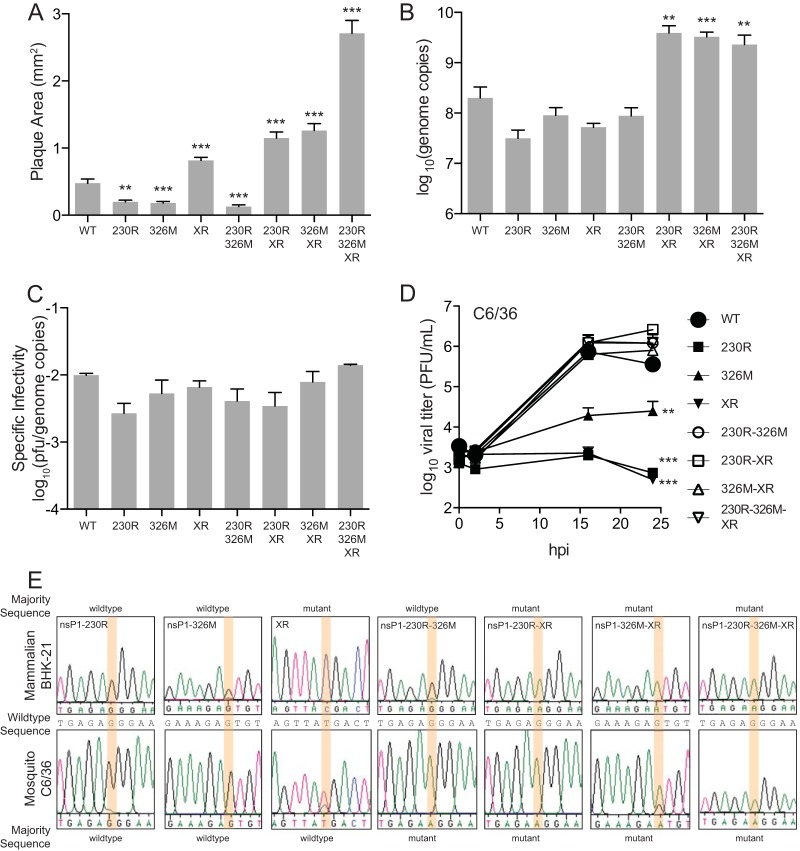
DFMO-resistant CHIKV exhibits fitness advantages *in vitro*. (A) CHIKV was diluted on confluent monolayers of Vero-E6 cells, and plaque size was measured after 72 h of infection. (B) BHK-21 cells were infected at an MOI of 0.1 with CHIKV and mutants and supernatant collected at 24 hpi. (C) CHIKV genome copies were measured by qRT-PCR and used to calculate specific infectivity of all viral mutants by dividing genome copies by the titers obtained from the same samples. (D) Aedes albopictus C6/36 cells were infected with CHIKV at an MOI of 0.1 and viral titers measured at the indicated times postinfection. (E) Representative chromatograms of sequencing reactions following mutant CHIKV passaged five times in BHK-21 (top panels) and C6/36 (bottom panels) cells in combination with wild-type CHIKV to measure fitness of mutants. Orange-highlighted regions correspond to positions of mutant nucleotides of interest, and the wild-type sequence is presented between the chromatograms. Whether the wild-type or mutant sequence presented as the majority is indicated above and below the chromatograms. *, *P* < 0.05; **, *P* < 0.01; ***, *P* < 0.001 (using Student's *t* test; *n* = 3). Error bars represent one standard error of the mean.

Because the three combination mutant viruses exhibited slightly enhanced replication kinetics (Fig. 2A), we considered whether these viruses may be more fit *in vitro*. To this end, we measured plaque sizes of these viruses following infection of confluent monolayers of Vero-E6 cells. We observed that all viruses with the XR mutation had larger plaques than wild-type CHIKV (Fig. 3A). In fact, the mutations in nsP1 contributed to a small-plaque phenotype. However, combining the nsP1 mutations with the XR mutation resulted in even larger plaques. We also investigated specific infectivity by measuring extracellular viral RNA levels and comparing them to viral titers as previously described (15). Consistent with enhanced replication, we observed enhanced viral RNA levels in the G230R-XR, V326M-XR, and G230R-V326M-XR mutants (Fig. 3B), though specific infectivity, measured by PFU per genome copy, was unchanged between the viral mutants (Fig. 3C).

Because arboviruses circulate between vertebrate and insect hosts, we also considered whether these viruses exhibit differences in growth kinetics in mosquito cells. To this end, we infected Aedes albopictus C6/36 cells with all viruses and measured titers over a time course (Fig. 3D). Interestingly, each of the single mutant viruses (G230R, V326M, and XR) exhibited severe defects in replication. However, combination of the mutations, including the G230R-V326M mutant, resulted in wild-type-level replication. Thus, these mutations may not emerge in nature due to the inability of the virus to replicate efficiently in the insect host with a single mutation.

Finally, to determine viral fitness, we used a competition assay between wild-type CHIKV and each of the mutants (16). Each virus was mixed with wild-type CHIKV at a 1:1 ratio and used to infect BHK-21 or C6/36 cells for 24 h. After five passages, supernatants were collected, and RNA was purified, reverse transcribed, and used for sequencing the mutations of interest. We found that the G230R, V326M, and G230R-V326M mutants were lost and the wild-type viral sequence was maintained in BHK-21 cells, indicative of decreased fitness compared to the wild-type sequence, and that all viruses with the XR mutation were present over the wild-type sequence, indicative of increased fitness (Fig. 3E). In C6/36 cells, in contrast, all single mutants were lost to the wild-type virus, and all viruses with combinations of mutations comprised the majority of the samples. The fitness decreases observed were not due to reversion, as all of the viruses were stable after serial passage on BHK-21 or C6/36 cells (data not shown). Together, these results suggest that our viral mutants have differential fitness and that all single mutants have reduced fitness in mosquito cells.

### Resistant viruses have enhanced replication *in vivo*.

**FIG 4:**
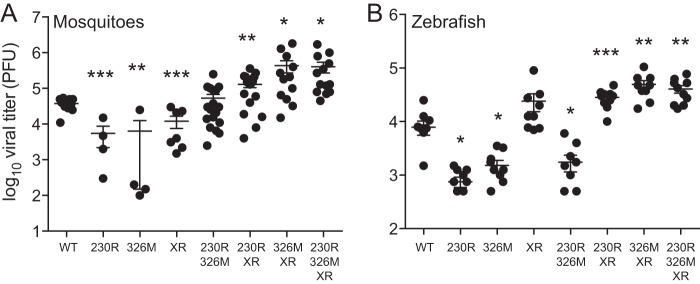
DFMO-resistant CHIKV exhibits enhanced replication *in vivo*. (A) Aedes albopictus mosquitoes were infected with a blood meal containing CHIKV and mutants, and viral titers in the whole mosquito were determined at 7 days postinfection. (B) Zebrafish were infected with CHIKV via intravenous injection and titers measured in whole fish 24 h later. *, *P* < 0.05; **, *P* < 0.01; ***, *P* < 0.001 (using Student's *t* test; *n* = 3). Error bars represent one standard error of the mean.

After observing that the DFMO-resistant viruses exhibited a fitness advantage *in vitro*, we explored whether this was true *in vivo* as well. We infected Aedes albopictus mosquitoes with all CHIKV mutants by providing 400 PFU in a blood meal. Seven days later, titers were determined from the whole body of the mosquito. As in our *in vitro* experiments, the single mutant viruses were attenuated in the mosquito (Fig. 4A). The G230R-XR, V326M-XR, and G230R-V326M-XR mutants, however, exhibited significantly higher titers in the mosquito. These results align with what we had observed *in vitro* in C6/36 cells.

Using a vertebrate model of CHIKV infection (17), we infected zebrafish with the CHIKV mutant viruses and measured viral titers at 48 hpi. As we had observed *in vitro*, the G230R, V326M, and G230R-V326M mutants exhibited reduced titers, while the G230R-XR, V326M-XR, and G230R-V326M-XR mutants had significantly higher titers than wild-type CHIKV (Fig. 4B).

### Analogous mutations in SINV confer resistance to polyamine depletion.

**FIG 5:**
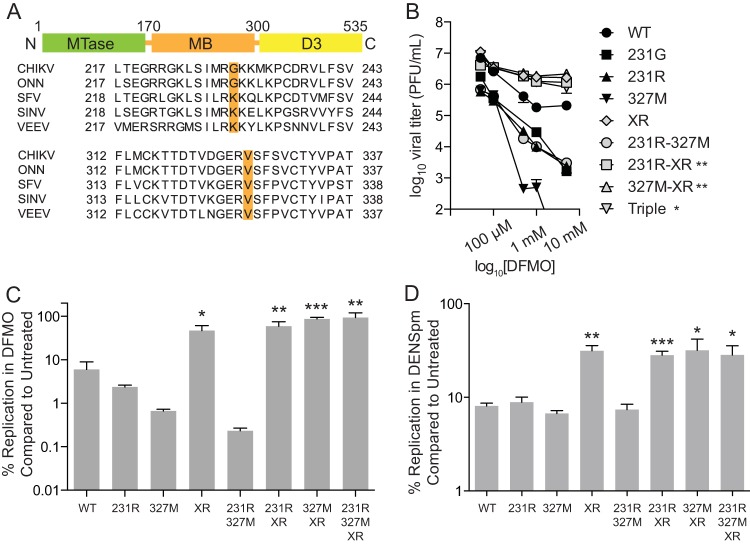
Analogous mutations in SINV confer resistance to polyamine depletion. (A) Schematic of nsP1 domains, including the methyltransferase (MTase) domain, the membrane binding (MB) domain, and domain 3 (D3). Sequence alignment of several alphaviruses is shown below with amino acids of interest highlighted. (B) BHK-21 cells were treated with increasing doses of DFMO for 4 days prior to infection with SINV. Titers were determined at 24 hpi. (C and D) Survival of SINV mutants was calculated by dividing the titers from DFMO-treated cells (C) or DENSpm-treated cells (D) by titers from untreated controls. *, *P* < 0.05; **, *P* < 0.01; ***, *P* < 0.001 (using Student's *t* test; *n* = 3). Error bars represent one standard error of the mean.

The nsP1 amino acid sequence, as well as the stop codon preceding nsP4, is shared with several alphaviruses (Fig. 5A). Thus, we considered whether the related alphavirus Sindbis virus (SINV) could exhibit resistance to polyamine depletion with the same mutations as CHIKV. Using site-directed mutagenesis, all mutations from CHIKV were transferred to SINV. BHK-21 cells were treated with increasing doses of DFMO and infected with the SINV mutants, and viral titers were determined at 24 h postinfection (hpi). We observed that the XR, 231R-XR, 327M-XR, and 231R-327M-XR mutants exhibited resistance to polyamine depletion (Fig. 5B). While approximately 10% of wild-type viruses survived DFMO treatment, compared to untreated controls, greater than 40% of mutant viruses containing the XR mutation survived 500 μM DFMO treatment (Fig. 5C). The same phenotype was observed with 50 μM DENSpm (Fig. 5D).

### Mutations enhance methylation of genomes and membrane binding.

**FIG 6:**
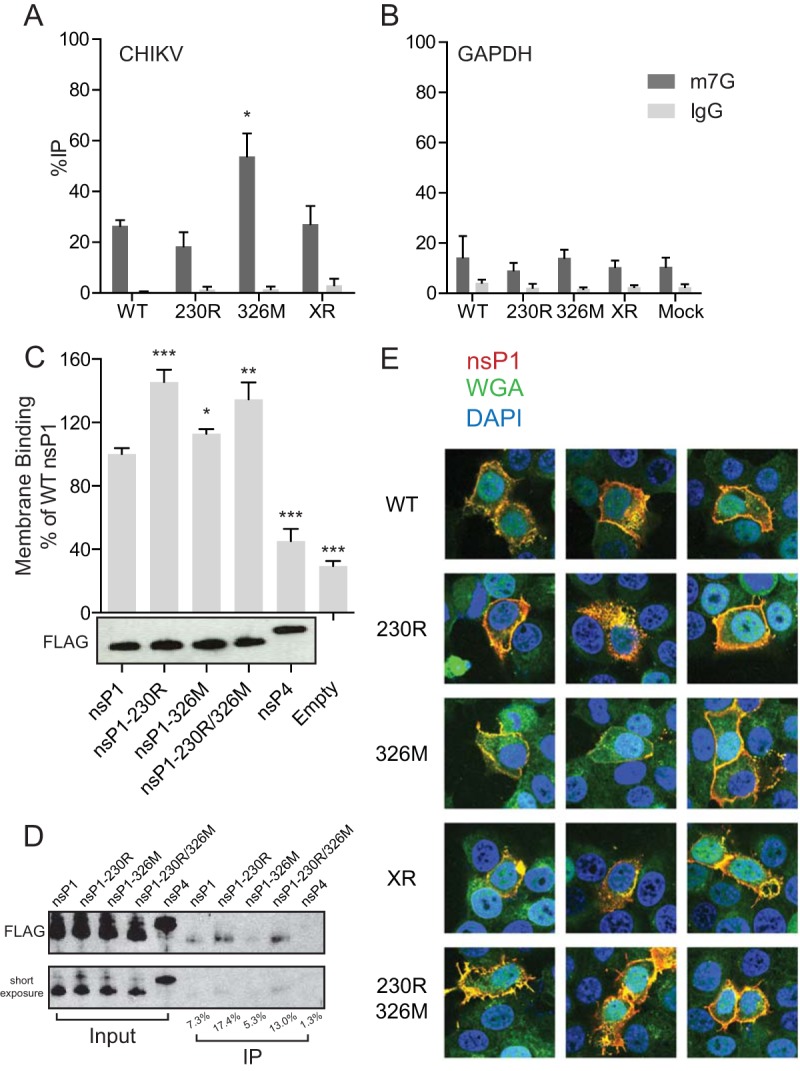
nsP1 mutations enhance genome capping and membrane binding. (A and B) RNA was isolated from infected cells and immunoprecipitated with antibodies against the m7G cap (dark gray bars) or IgG (light gray bars). Immunoprecipitated CHIKV genomes (A) and cellular GAPDH mRNA (B) were quantified by qRT-PCR and compared to input RNA. (C) nsP1 and nsP4 were transcribed and translated *in vitro*, and membrane binding activity was measured by ELISA. Membrane binding ability was compared to that of wild-type nsP1 protein. Expression of proteins was assessed by Western blotting against a FLAG epitope on each protein (a representative blot is shown below the graph). (D) nsP1 and nsP4 were mixed with membranes from BHK-21 cells and cosedimented. Membrane binding was measured by Western blotting against the FLAG epitope and quantified (bottom panel). (E) Wild-type and mutant nsP1 proteins were visualized via indirect immunofluorescence by staining for nsP1 in infected HeLa cells. Cells were infected for 16 h and then fixed and stained for nsP1 (red), wheat germ agglutinin (WGA) (green), and DAPI (blue). Three representative images are displayed for each virus. *, *P* < 0.05; **, *P* < 0.01; ***, *P* < 0.001 (using Student's *t* test; *n* = 3). Error bars represent one standard error of the mean.

To address the functional implications of these mutations, we looked into how they were impacting viral processes. nsP1 is a multifunctional enzyme, with GTPase, guanylyltransferase, and methyltransferase activities, functioning to cap viral genomes for efficient translation. Because the V326M mutation was present in the as-yet-undescribed domain 3 (Fig. 5A) (18), we chose to focus on what impact this mutation would have on viral genome capping. BHK-21 cells were infected at an MOI of 0.1 with the single-mutation viruses, which have the same replication kinetics (Fig. 2A). At 16 hpi, cells were harvested and total RNA purified. The purified RNA was subjected to immunoprecipitation with an antibody against the m^7^G cap (19), present on capped CHIKV genomes and cellular mRNAs, or with nonspecific IgG. Coimmunoprecipitated RNA was reverse transcribed and quantitated via quantitative PCR (qPCR) with primers against CHIKV genomes (Fig. 6A) and cellular GAPDH (glyceraldehyde-3-phosphate dehydrogenase) (Fig. 6B). We observed that in V326M CHIKV infection, the percent immunoprecipitated was significantly higher than in wild-type, G230R mutant, or XR mutant infection. The assay was specific, as only very small RNA amounts were recovered with control IgG. Coimmunoprecipitating GAPDH sequences were not significantly different across the samples. Thus, a significantly higher proportion of viral genomes coimmunoprecipitate with the m7G antibody in V326M CHIKV infection, suggesting that this mutation may either enhance capping or alter cap structure to allow enhanced coimmunoprecipitation.

The G230R mutation is located near a patch of amino acids that have been implicated in membrane binding (20, 21); thus, we investigated whether this mutation impacts the ability of nsP1 to bind membranes. Wild-type and mutant nsP1 protein, as well as nsP4 protein, were generated via *in vitro* transcription (IVT) and translation and then subjected to a membrane enzyme-linked immunosorbent assay (ELISA) to measure the ability of these proteins to bind to membranes. Crude membrane extracts were prepared from BHK-21 cells and used to coat ELISA plates. The ability of the nsP1 proteins to bind to these membranes was measured by detecting the FLAG epitope on nsP1 (or nsP4, our control) via ELISA. We observed that the G230R mutant, as well as the G230R-V326M double mutant, exhibited enhanced binding to these membranes, with levels 20 to 50% higher than that of wild-type nsP1 (Fig. 6C). A Western blot of the FLAG epitope tag on each of the proteins demonstrated that each protein was expressed well, suggesting that enhanced binding was not due to a significant increase in the amount of 230R nsP1 or 230R-326M nsP1 (Fig. 6C, lower panel). Additionally, our control protein, nsP4, did not bind membranes, as binding levels were close to those in background (empty) samples. To confirm these results, *in vitro*-transcribed and translated nsP1 and nsP4 were combined with BHK-21-derived membranes as in our membrane ELISA, incubated on ice, and sedimented via centrifugation. Cosedimentation of nsP1 with the membranes was detected via Western blotting against the FLAG epitope. As with the membrane ELISA, the G230R and G230R-V326M nsP1 proteins exhibited enhanced sedimentation compared to wild-type nsP1 (Fig. 6D), suggesting that these constructs bound to the membranes better and, thus, cosedimented. Together, these results suggest that the G230R mutation functions to enhance membrane binding of nsP1.

To determine whether the nsP1 mutations may alter the localization of nsP1 during infection, we visualized nsP1 via indirect immunofluorescence. HeLa cells were infected at an MOI of 0.1 with the wild-type, nsP1-230R, nsP1-326M, XR, and nsP1-230R-326M viruses for 16 h and subsequently fixed and stained using an nsP1-specific antibody, as well as wheat germ agglutinin to label cellular membranes and DAPI (4′,6′-diamidino-2-phenylindole) to visualize cellular DNA. In each infection, we noted overlapping signal between nsP1 and wheat germ agglutinin, suggesting membrane association. However, we also noted heterogeneity in staining patterns in each of the infections. Thus, we were unable to determine whether nsP1 changes its localization or has enhanced membrane association via indirect immunofluorescence staining. This could be because either the immunofluorescence is not optimized for this phenotype or because our *in vitro* results do not carry to the context of infection.

### Mutation of the opal stop codon to arginine enhances downstream translation.

**FIG 7:**
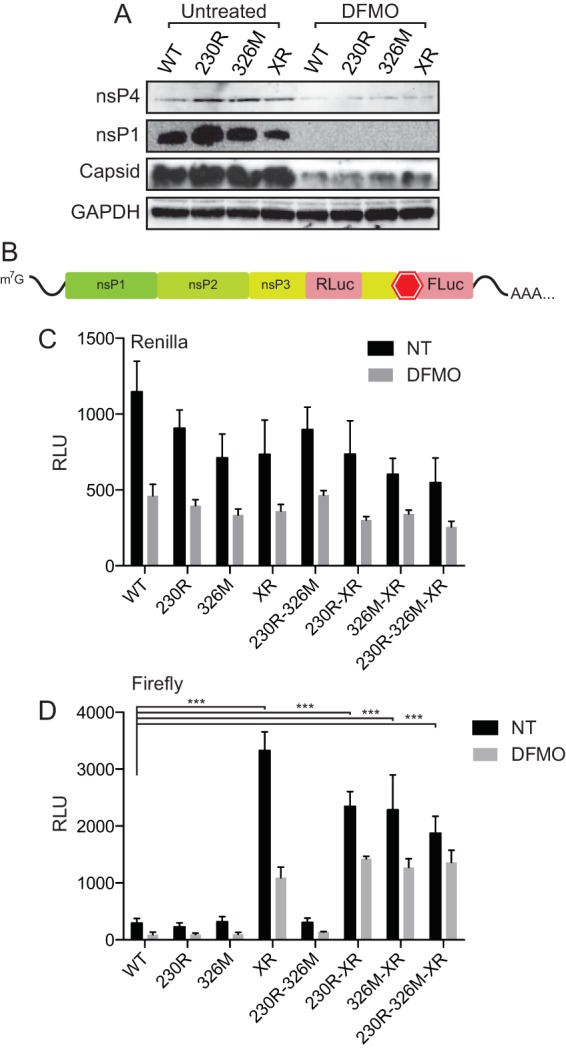
Mutation of the opal stop codon enhances downstream protein expression. (A) BHK-21 cells left untreated or treated with 500 μM DFMO were infected at an MOI of 0.1 for 24 h, and viral protein levels for nsP1, nsP4, and capsid were analyzed by Western blotting. (B) Schematic of the luciferase construct used to investigate the impact of the stop codon after nsP3. Renilla luciferase (RLuc) is located prior to the stop codon, and firefly luciferase (FLuc) is after the stop codon. (C) Renilla luciferase activity in 293T cells transfected with RNA generated from the construct shown in panel B, either left untreated (black bars) or treated with 500 μM DFMO (gray bars). (D) Firefly luciferase activity in 293T cells similarly transfected and treated. ***, *P* < 0.001 (using Student's *t* test; *n* = 3). Error bars represent one standard error of the mean.

The function of the stop codon in the context of alphavirus infection is not entirely understood, though it has appeared in other screens for drug-resistant CHIKV (22). To determine whether the stop codon modified the amount of nsP4 within the infected cell, we infected BHK-21 cells at an MOI of 0.1 for 16 h and then collected lysates for Western blot analysis of viral proteins. We noted no significant difference in nsP4 levels in either untreated or DFMO-treated cells, commensurate with levels of nsP1 and capsid (Fig. 7A), suggesting that the stop codon does not affect steady-state levels of nsP4, as detected by Western blot analysis.

To more closely monitor expression of proteins downstream of this viral stop codon, we employed a more sensitive luciferase assay. In this construct, Renilla luciferase is placed within the nsP3-coding sequence and firefly luciferase is placed directly after the opal stop codon (Fig. 7B). This molecule is nonreplicating, due to the lack of nsP4, so any measurement of luciferase activity is a measurement of translation of the construct but not replication. RNA was generated from this construct along with all of the combinations of viral mutants. This RNA was transfected into untreated or DFMO-treated 293T cells (to increase transfection efficiency and signal), and Renilla and firefly luciferase activities were measured 24 h later. We observed that Renilla activity was not significantly different among any of the mutants and that DFMO significantly reduced this activity (Fig. 7C), in line with our previous findings that DFMO reduces viral translation (9). However, when we measured firefly luciferase activity, we noted that all viral mutants with the XR mutation had significantly increased activity (Fig. 7D), suggesting that removal of this stop codon enhances downstream translation. We did not see any significant differences when the XR mutation was combined with either nsP1 mutation, however.

## DISCUSSION

The generation of resistance to antiviral therapies is a common problem in the fight against infectious diseases. Because viruses rapidly mutate, the development of antiviral resistance may be inevitable, but by understanding the outcomes of antiviral resistance, we may learn more about viral replication and better design antiviral therapy regimens, such as through combination therapy. Although DFMO targets a host enzyme and disrupts the host polyamine pools, we have been able to generate CHIKV that is resistant to polyamine depletion. This resistance is not in itself surprising; however, that the described mutations must cooperate to generate resistance is less commonly described.

The mutations that arise over the course of passaging in polyamine-depleted cells enhance viral replication *in vitro* and *in vivo*, though only when in combination. Curiously, individual mutations do not contribute to resistance to polyamine depletion and lead to a fitness decrease. This may preclude the appearance of the double or triple mutants, with enhanced fitness, in nature. However, we do not precisely know the timing of the mutations and whether they may have emerged linked on the same viral genome. If the mutations were to emerge individually, the fitness disadvantage for each of these single mutations in mosquitoes would provide a significant hurdle for the emergence of the double or triple mutants with enhanced fitness. Thus, despite our findings that CHIKV is able to attain resistance to DFMO-mediated polyamine depletion, the virus may not be able to generate these mutations in nature due to the associated fitness costs *in vivo*.

We were able to transfer the mutations observed from CHIKV into the related virus SINV and also to observe DFMO resistance in SINV. Curiously, however, SINV required only that the stop codon be changed to an arginine for DFMO resistance. This observation may be due to the basic lysine already present at amino acid 231 in SINV, whereas CHIKV has a glycine at amino acid 230. For CHIKV, a basic arginine residue at this position confers DFMO resistance (in combination with the XR mutation); thus, SINV may need only this single XR mutation to develop resistance. In fact, we have observed that SINV is less sensitive to polyamine depletion than CHIKV: SINV titers are reduced approximately 10-fold, while CHIKV titers are reduced 1,000-fold, with 500 μM DFMO treatment. Perhaps this differential sensitivity is due to amino acid changes already present within the SINV genome.

nsP1 contains enzymatic functions that contribute to viral infection through replication complex anchoring through the membrane binding domain as well as through the capping of the viral genome via methyltransferase and guanylyltransferase activity. These functions contribute to viral translation, a process that requires polyamines (9). Therefore, the appearance of mutations in this region fits with a role for polyamines in translation. How these mutations by themselves are detrimental for viral replication *in vivo* is unclear. Perhaps the mutations are involved in pathogenesis or replication in specific cell types not recapitulated *in vitro*, an area that requires further in-depth investigation. However, when the mutations are combined, CHIKV gains fitness both *in vitro* and *in vivo*, as nsP1's function is enhanced.

Chikungunya virus, as well as several related alphaviruses, carries an opal stop codon between the nsP3 and nsP4 genes (23). The precise function of this stop codon is unknown, though hypotheses have considered that it is important in the regulation of nsP4 protein levels (23, 24). Previous studies have demonstrated that O'nyong-nyong virus with its stop codon mutated to an arginine is attenuated in mosquitoes (24), similar to our results with CHIKV. Further, this mutation appeared in another study upon inhibition of the sodium-potassium ATPase (22). Our data suggest that the presence of the stop codon was detrimental to virus replication and protein expression in polyamine-depleted cells as well as in insects. Further, changing this stop codon to an arginine codon significantly enhanced downstream protein expression. Precisely why this mutation is detrimental in insects remains to be understood. Polyamines have been implicated in ribosomal slippage and bypassing stop codons in the context of cellular mRNAs (25–28), and CHIKV may also rely on polyamines to bypass its internal stop codon. Because polyamines are required for optimal polymerase activity, the virus may overcome this deficiency by simply producing more polymerase, via mutation of the opal stop codon. Others have found a common stem-loop that facilitates stop codon readthrough in alphaviruses (29). Interestingly, polyamines are implicated in RNA structure and may impact stop codon readthrough by altering this stem-loop structure. Future work will address this important question.

Together, our data present novel means by which chikungunya virus responds to a host-targeted antiviral. Previously we suggested polyamine depletion as a means to treat viral infection clinically (10), and the availability and tolerability of DFMO suggest that the drug could combat viral infection (8). Given the response of CHIKV to DFMO treatment through nsP1 and stop codon mutations, a better antiviral solution could exist in combination therapy, combining DFMO with other antiviral agents to reduce the likelihood of generating resistance. Because DFMO resistance requires at least two independent mutations, the likelihood of observing resistance *in vivo* is reduced, though this likelihood could be reduced even further with combination therapy.

## MATERIALS AND METHODS

### Cell culture.

Mammalian 293T, HeLa, and BHK-21 cells were maintained at 37°C in 5% CO_2_ in Dulbecco's modified Eagle's medium (DMEM) (Life Technologies) supplemented with bovine serum and penicillin-streptomycin. Aedes albopictus C6/36 cells were maintained at 28°C in L-15 medium with bovine serum and penicillin-streptomycin.

### Drug treatment.

DFMO (Tebu Bio) and DENSpm (Tocris Bioscience) were diluted to a 100× solution (50 mM and 10 mM, respectively) in sterile water and stored at 4°C until use. For DFMO treatments, cells were trypsinized (Life Technologies) and reseeded with fresh medium supplemented with 2% serum. Following overnight attachment, cells were treated with 500 μM DFMO. Cells were incubated with the drug for 96 h to allow for depletion of polyamines in BHK-21 cells and for 48 h in 293T cells. For DENSpm treatment, cells were treated 16 h prior to infection at the indicated concentrations. During infection, medium was cleared from the cells and saved. The same medium containing DFMO or DENSpm was then used to replenish the cells following infection.

### Infection and enumeration of viral titers.

CHIKV (La Réunion strain, 06-049 AM258994) and SINV (pTE/3′2J strain) were derived from the first passage of virus in BHK cells. Viral stocks were maintained at −80°C. For all infections, DFMO and DENSpm were maintained throughout infection as designated.

For infection, virus was diluted in serum-free DMEM at a multiplicity of infection (MOI) of 0.1, unless otherwise indicated. Viral inoculum was overlaid on cells for 30 min, virus was then cleared from the cells, and the cells were washed three times with phosphate-buffered saline (PBS). The medium was replenished as described above.

Supernatants were collected from infections at 24 hpi, unless otherwise indicated. Dilutions of cell supernatant were prepared in serum-free DMEM and used to inoculate confluent monolayers of Vero-E6 cells for 30 min to 1 h at 37°C. Cells were then overlaid with 0.8% agarose in DMEM containing 1.6% bovine serum. CHIKV samples were incubated for 72 h and SINV samples for 48 h. Following incubation, cells were fixed with 4% formalin and revealed with crystal violet solution (10% crystal violet [Sigma-Aldrich], 20% ethanol). Plaques were enumerated and used to back calculate the number of PFU per milliliter of collected volume.

### RNA purification and cDNA synthesis.

Medium was cleared from cells and TRIzol reagent (Sigma-Aldrich) directly added. Lysate was then collected and RNA purified according to the manufacturer's protocol. Purified RNA was DNase treated (Ambion) and subsequently used for cDNA synthesis using Maxima H Minus reverse transcriptase (Life Technologies), according to the manufacturer's protocol, with 500 ng of RNA and random hexamer primers.

### Viral genome quantitation.

Following cDNA synthesis, quantitative reverse transcription-PCR (qRT-PCR) was performed using StepOnePlus (Applied Biosystems, Norwalk, CT) and SYBR green master mix (Life Technologies). Samples were held at 95°C for 30 s prior to 40 cycles of 95°C for 10 s and 60°C for 15 s. Primers for CHIKV targeted residues 2248 to 2480 in the viral genome (forward, 5′-CAC-CGA-CGT-GAT-GAG-AC-3; reverse, 5′-CAT-ATT-GAA-GAA-GCC-GCA-C-3′) Primers against GAPDH (forward, 5′-GGT-GTG-AAC-CAT-GAG-AAG-TAT-GA-3′; reverse, 5′-GAG-TCC-TTC-CAC-GAT-ACC-AAA-G-3′) were used to normalize to total RNA using the Δ*C_T_* method.

### Western blots.

Samples diluted in Laemmli buffer were run on 12% polyacrylamide gels and transferred using the Mini Trans-Blot cell (Bio-Rad). Transferred proteins on polyvinylidene difluoride (PVDF) membranes (GE Healthcare) were blocked in Tris-buffered saline containing 0.2% Tween 20 (0.2% TBST) with 5% bovine serum albumin (BSA). Membranes were probed with primary antibody for GAPDH (1:5,000; Santa Cruz Biotechnology), FLAG (1:1,000; Sigma-Aldrich), CHIKV nsP4 (1:2,000), CHIKV nsP1 (1:5,000), and CHIKV capsid (1:10,000) overnight at 4°C. Membranes were washed three times with 0.2% TBST and then incubated with secondary antibody (1:10,000; GE Healthcare) for 45 min at room temperature. Membranes were washed again three times with 0.2% TBST and then treated with SuperSignal West Pico chemiluminescent detection reagent (Thermo Scientific). Membranes were exposed to Amersham Hyperfilm (GE Healthcare) and developed.

### DFMO passages.

BHK-21 cells were treated with 500 μM DFMO, or left untreated, for 4 days. For the first passage, cells were infected at an MOI of 0.1, and supernatants were collected at 24 hpi. For blind passages, 5 μl of supernatant was used to inoculate subsequent passages. For fixed-MOI passages, viruses titers were determined, and the next passage was infected at an MOI of 0.1.

### Sequencing and determination of viral mutants.

After 10 fixed-MOI passages or 21 blind passages, supernatants were collected in TRIzol and RNA purified and reverse transcribed as described above. Genomes were amplified using the PCR primers described by Schuffenecker et al. (30). Amplified DNA was Sanger sequenced to cover the full viral genome, and mutations were identified by alignment and comparison of starting virus to passaged virus (DNAStar Lasergene). Mutations were identified if they were the majority of the signal in the Sanger sequencing reaction. Chromatograms were used to confirm the presence of the mutation within the samples.

### DFMO sensitivity assays.

BHK-21 cells were treated with DFMO at doses of 5 μM to 5 mM for 4 days prior to infection with CHIKV or SINV at an MOI of 0.1. At 24 hpi, supernatants were collected and titers were determined.

### Site-directed mutagenesis.

Wild-type clones of CHIKV, CHIKV luciferase replicon, and SINV were mutagenized with the QuikChange II XL site-directed mutagenesis kit (Agilent). Mutagenic primers for CHIKV targeted nsP1 G230R (forward, 5′-CAA-GTT-GTC-TAT-TAT-GAG-AAG-GAA-AAA-GCT-AAA-ACC-G-3′; reverse, 5′-CGG-TTT-TAG-CTT-TTT-CCT-TCT-CAT-AAT-AGA-CAA-CTT-G-3′), V326M (forward, 5′-CGG-TTG-ACG-GCG-AAA-GAA-TGT-CAT-TCT-CGG-TGT-GC-3′; reverse, 5′-GCA-CAC-CGA-GAA-TGA-CAT-TCT-TTC-GCC-GTC-AAC-CG-3′), and the opal stop codon (forward, 5′-GAC-ACG-GAC-GAC-GAG-TTA-CGA-CTA-GAC-AGG-GCA-GG-3′; reverse, 5′-CCT-GCC-CTG-TCT-AGT-CGT-AAC-TCG-TCG-TCC-GTG-TC-3′). Mutagenic primers for SINV targeted nsP1 K231R (forward, 5′-GGA-AAA-TTG-TCG-ATA-ATG-AGG-AGG-AAG-GAG-TTG-AAG-C-3′; reverse, 5′-GCT-TCA-ACT-CCT-TCC-TCC-TCA-TTA-TCG-ACA-ATT-TTC-C-3′), V327M (forward, 5′-GGA-GAA-CGG-GTA-TCG-TTC-CCT-ATG-TGC-ACG-TAC-ATC-C-3′; reverse, 5′-GGA-TGT-ACG-TGC-ACA-TAG-GGA-ACG-ATA-CCC-GTT-CTC-C-3′), and the opal stop codon (forward, 5′-GCA-GGA-GGA-CTG-AAT-ACC-GAC-TAA-CCG-GGG-TAG-G-3′; reverse, 5′-CCT-ACC-CCG-GTT-AGT-CGG-TAT-TCA-GTC-CTC-CTG-C-3′). Mutagenic PCR conditions were as follows: 95°C for 60 s; 18 cycles of 95°C for 50 s, 60°C for 50 s, and 68° for 13 min; and 68°C for 7 min. PCR products were DpnI treated for 3 h at 37°C and then transformed into XL-10 Gold cells. The mutant plasmid was confirmed by Sanger sequencing.

The CHIKV luciferase replicon was altered to remove nsP4, rendering it incapable of replication, and adjoining firefly luciferase immediately downstream of the stop codon following nsP3. These constructs were generated from the mutated replicon constructs using forward primer 5′-GAG-AAT-ATA-TAC-CCA-CCT-GCC-CTG-3′ and reverse primer 5′-CAG-GTG-GGT-ATA-TAT-TCT-CGG-AAG-ATG-CCA-AAA-ACA-TTA-AGA-AGG-G-3′ and using *in vivo* assembly (IVA) cloning as described previously (31).

### IVT and generation of mutant viruses.

CHIKV was generated from the La Reunion strain 06-049 infectious clone, and its expression plasmid was linearized with NotI. SINV was generated from the pTE/3′2J strain and linearized with XhoI. CHIKV replicons were linearized with NotI. Linearized DNA fragments were purified by phenol-chloroform extraction. Ethanol-precipitated DNA was then used for *in vitro* transcription (IVT) using the SP6 mMessage mMachine kit (Ambion). RNAs were purified by phenol-chloroform extraction, ethanol precipitation, quantified, and diluted to 1 μg/μl for storage at −80°C.

RNA was transfected into BHK-21 cells using Lipofectamine 2000 (Thermo Fisher), and supernatants were collected upon full cytopathic effect to generate stocks. RNA was extracted from supernatant and reverse transcribed, and the presence of mutations was verified by Sanger sequencing.

### *In vitro* transcription-translation (IVTT).

Plasmids encoding nsP1 and mutants, along with nsP4, were combined with rabbit reticulocyte lysate in the TNT Quick Couple Transcription/Translation system (Promega) according to the manufacturer's instructions. Protein was expressed for 3 h prior to Western blot analysis or membrane immunoprecipitation.

### Plaque size measurement.

The titer of mutant CHIKV was determined on Vero-E6 cells as described above. After development of plaques, images were taken of plates and distances across plaques measured using ImageJ. Distances were converted to millimeters by comparing measurements to a ruler from the same image. Distances were converted into plaque area. At least 20 plaques were measured per virus.

### Specific infectivity measurement.

BHK-21 cells were infected at an MOI of 0.1 with CHIKV mutants. At 16 hpi, supernatants were collected and titers were determined as described above. Supernatants were also combined with TRIzol, RNA purified, and reverse transcribed. CHIKV genomes were quantified by qPCR using primers specific to CHIKV and compared to a standard curve using the cloned viral genome.

### Stability and fitness assays.

To measure stability of the mutations, BHK-21 and C6/36 cells were infected at an MOI of 0.1 with all viral mutants for 24 h. Virus was then passed to new cells by transferring 10 μl of supernatant. After five passages, RNA was extracted and purified from supernatants and reverse transcribed. Sanger sequencing was used to determine whether mutations were stable over the passages by looking at the chromatograms and determining presence or absence of the mutant nucleotide. Fitness assays were similarly performed, but BHK-21 and C6/36 cells were infected at an MOI of 0.1 with an equal combination of wild-type and mutant CHIKV and passaged five times in the respective cell line. Fitness was determined by Sanger sequencing and analysis of the chromatogram to determine if the wild-type or mutant nucleotide was most abundant within the sample.

### Mosquito infection.

A laboratory colony of Aedes albopictus was used for mosquito infections 8 to 13 generations after it was collected in 2011 in Phu Hoa, Ben Cat District, Binh Duong Province, Vietnam. One day before mosquito infection, female mosquitoes were sorted and starved overnight. On the day of infection, 7-day-old female mosquitoes were allowed to feed on prewashed rabbit blood meals containing 5 mM ATP and 400 PFU/ml CHIKV for 30 min at 37°C. After blood feeding, engorged females were sorted into individual boxes (20 mosquitoes/box) and incubated at 28°C with 70% humidity. Mosquitoes were fed on 10% sucrose for the duration of the experiment. At the end of incubation, the mosquitoes were placed into 2-ml round-bottom tubes containing 200 μl of PBS and a steel ball (Qiagen). Mosquitoes were ground in an MM300 homogenizer (Qiagen) at 30 shakes per s for 2 min. Viral titers were determined by plaque assay.

### Zebrafish infection.

Zebrafish larvae were inoculated by microinjection of ∼10^2^ PFU CHIKV (∼1 nl of supernatant from infected BHK cells, diluted to 10^8^ PFU/ml) intracerebrally. Larvae were then distributed in 24-well culture plates, kept at 28°C, and regularly inspected with a stereomicroscope. Zebrafish were anesthetized at 2 days postinfection, frozen, and homogenized. Viral titers were determined as described above.

### Methylated genome immunoprecipitation.

BHK-21 cells were infected at an MOI of 1 with CHIKV, and cells were collected in TRIzol at 8 hpi. RNA was purified, and 1/6 of the total volume of RNA was combined with 1 μl αm7G-cap antibody or 1 μl nonspecific IgG, 20 μl protein A/G-agarose beads, and 900 μl PBS. The mixture was incubated while rotating overnight at 4°C. The beads were washed five times with cold PBS containing 1% Tween 20. Immunoprecipitated RNA and input RNA (1/6 of the total volume of RNA purified) were reverse transcribed and analyzed by qPCR for CHIKV (forward, 5′-CAC-CGA-CGT-GAT-GAG-AC-3; reverse, 5′-CAT-ATT-GAA-GAA-GCC-GCA-C-3′) and GAPDH (forward, 5′-GGT-GTG-AAC-CAT-GAG-AAG-TAT-GA-3′; reverse, 5′-GAG-TCC-TTC-CAC-GAT-ACC-AAA-G-3′) with SYBR green (Life Technologies) and the StepOne system (Thermo Fisher). Percent immunoprecipitation was calculated by comparing threshold cycle (*C_T_*) values for input and immunoprecipitated RNAs.

### Membrane binding ELISA.

BHK-21 cells were grown to confluence in T175 flasks and subsequently trypsinized, washed once in cold PBS, and resuspended in swelling buffer (10 mM Tris-HCl [pH 7.4], 10 mM NaCl, 1.5 mM MgCl, and protease inhibitor). After a 15-min incubation on ice, cells were Dounce homogenized 30 times. Lysed cells were spun at 500 × *g* for 5 min and supernatant collected. The supernatant was then centrifuged at 13,000 × *g* for 5 min. The membrane pellet was collected and resuspended in storage buffer (10 mM Tris-HCl [pH 7.4], 10 mM NaCl, 250 mM sucrose, and protease inhibitor) to wash. The membranes were then resuspended in 75 μl storage buffer per confluent flask. Half of the membranes from a flask were then combined with PBS to fill a 96-well plate. Plates were then maintained at room temperature to allow evaporation of PBS, leaving membranes attached to the plates. One microliter of IVTT protein from nsP1 or nsP4 was combined with 20 μl PBS, added to the plates, and incubated at 37°C for 2 h. Control wells were incubated only with 20 μl of PBS. Following incubation, plates were washed three times with PBS containing 0.2% Tween 20. Antibody against the FLAG epitope was combined with PBS for a final concentration of 0.1 μl antibody per well and incubated in wells for 2 h. The plate was again washed three times with 0.2% PBS–Tween. Secondary antibody (goat anti-mouse) was then incubated in wells at a final concentration of 0.1 μl antibody per well for 30 min. Wells were washed five times with 0.2% PBS–Tween before development with 70 μl tetramethylbenzidine (TMB) (Thermo-Fisher) for 5 min. To stop the reaction, 70 μl H_2_SO_4_ was added. The plate was then read at 450 nm. The percentage bound was calculated by comparing the absorbance reading for mutant nsP1, nsP4, or empty wells to that for wild-type nsP1.

### Membrane binding Western blot assay.

Membranes from BHK-21 cells were prepared as described above. Ten microliters of membranes were combined with 5 μl of *in vitro*-generated protein and 900 μl PBS, and the mixture was incubated, rotating, overnight at 4°C. Membranes were then precipitated by centrifugation at 11,000 rpm for 1 min. Membranes were washed six times with PBS containing 0.5% Tween 20. Following the final wash, membranes were resuspended in Laemmli buffer and analyzed by Western blotting.

### Immunofluorescence.

HeLa cells were seeded to coverslips and infected at an MOI of 0.1 with CHIKV and mutants. At 16 hpi, medium was cleared, coverslips were washed twice with PBS, and cells were fixed with 4% formalin for 15 min at room temperature. Coverslips were then washed three times with PBS to remove formalin. Samples were then blocked using PBS supplemented with 5% bovine serum albumin for 30 min at room temperature. Primary staining was done for 2 h at room temperature using antibodies against nsP1 (1:1,000), wheat germ agglutinin (1:1,000; Millipore), and DAPI (1:1,000; Thermo Scientific) diluted in BSA-PBS. Following three washes in TBS with 0.2% Tween 20, coverslips were stained with anti-rabbit and -mouse secondary antibodies (1:1,000; Invitrogen, Carlsbad, CA) diluted in BSA-PBS. Three final washes were performed, and coverslips were mounted using Vectashield (Vector Laboratories). Images were acquired with a Leica SPE inverted confocal microscope. Image processing was carried out with Adobe Photoshop CS6 software.

### Luciferase assays.

293T cells were treated with 500 μM DFMO for 2 days and subsequently transfected using Lipofectamine 2000 (Life Technologies) with CHIKV replicon RNA (32). For luciferase assays, cells were combined with Renilla (Renilla-Glo; Promega) or firefly (Bright-Glo; Promega) luciferase substrate at 24 h posttransfection. Luciferase assays were performed according to the manufacturer's recommendations (Promega), and results were measured using the Wallac 1420 instrument (PerkinElmer).

### Statistical analysis.

Prism 6 (GraphPad) was used to generate graphs and perform statistical analysis. For all analyses, the one-tailed Student's *t* test was used to compare groups, with α = 0.05, unless otherwise noted. IC_50_ values were calculated from GraphPad Prism via the nonlinear regression analysis. Analyses of variance (ANOVAs) were similarly performed in GraphPad Prism via grouped analysis. Significance was considered if the appropriate factor was deemed significant (*P* < 0.05).
